# The relations between sleep, time of physical activity, and time outdoors among adult women

**DOI:** 10.1371/journal.pone.0182013

**Published:** 2017-09-06

**Authors:** Kate Murray, Suneeta Godbole, Loki Natarajan, Kelsie Full, J. Aaron Hipp, Karen Glanz, Jonathan Mitchell, Francine Laden, Peter James, Mirja Quante, Jacqueline Kerr

**Affiliations:** 1 School of Psychology and Counselling, Queensland University of Technology, Brisbane, Queensland, Australia; 2 Department of Family Medicine & Public Health, University of California, San Diego, La Jolla, California, United States of America; 3 Department of Parks, Recreation, and Tourism Management, North Carolina State University, Raleigh, North Carolina, United States of America; 4 Center for Geospatial Analytics, North Carolina State University, Raleigh, North Carolina, United States of America; 5 Center for Human Health and the Environment, North Carolina State University, Raleigh, North Carolina, United States of America; 6 Perelman School of Medicine and School of Nursing, University of Pennsylvania, Philadelphia, Pennsylvania, United States of America; 7 Division of Gastroenterology, Hepatology and Nutrition, Children’s Hospital of Philadelphia, Philadelphia, Pennsylvania, United States of America; 8 Departments of Environmental Health and Epidemiology, Harvard T.H. Chan School of Public Health, Boston, Massachusetts, United States of America; 9 Channing Division of Network Medicine, Brigham and Women’s Hospital and Harvard Medical School, Boston, Massachusetts, United States of America; 10 Division of Chronic Disease Research Across the Lifecourse (CoRAL), Department of Population Medicine, Harvard Medical School and Harvard Pilgrim Health Care Institute, Boston, Massachusetts, United States of America; 11 Division of Sleep and Circadian Disorders, Departments of Medicine and Neurology, Brigham and Women’s Hospital, Harvard Medical School, Boston, Massachusetts, United States of America; 12 Department of Neonatology, University of Tuebingen, Tuebingen, Germany; University of Rome Tor Vergata, ITALY

## Abstract

Physical activity and time spent outdoors may be important non-pharmacological approaches to improve sleep quality and duration (or sleep patterns) but there is little empirical research evaluating the two simultaneously. The current study assesses the role of physical activity and time outdoors in predicting sleep health by using objective measurement of the three variables. A convenience sample of 360 adult women (mean age = 55.38 ±9.89 years; mean body mass index = 27.74 ±6.12) was recruited from different regions of the U.S. Participants wore a Global Positioning System device and ActiGraph GT3X+ accelerometers on the hip for 7 days and on the wrist for 7 days and 7 nights to assess total time and time of day spent outdoors, total minutes in moderate-to-vigorous physical activity per day, and 4 measures of sleep health, respectively. A generalized mixed-effects model was used to assess temporal associations between moderate-to-vigorous physical activity, outdoor time, and sleep at the daily level (days = 1931) within individuals. There was a significant interaction (*p* = 0.04) between moderate-to-vigorous physical activity and time spent outdoors in predicting total sleep time but not for predicting sleep efficiency. Increasing time outdoors in the afternoon (versus morning) predicted lower sleep efficiency, but had no effect on total sleep time. Time spent outdoors and the time of day spent outdoors may be important moderators in assessing the relation between physical activity and sleep. More research is needed in larger populations using experimental designs.

## Introduction

Sleep disturbances are highly prevalent in the US [1] with large proportions of adults reporting sleep difficulties including initiating and maintaining sleep [2, 3]. Research has identified significant changes in sleep structure during the aging process, including changes in the proportion of sleep spent in different phases of sleep [3, 4] and increased sleep disturbances [4, 5]. The increase of sleep disturbances during the aging process may reflect changes in circadian rhythms that occur in aging, alongside other health and lifestyle factors such as increases in comorbidities (e.g. chronic pain, obesity, neurological disorders) and use of pharmacological interventions that may affect sleep [3].

Sleep accounts for nearly one-third of daily activity [6] and cross-sectional studies have demonstrated that shortened sleep duration is linked to obesity, type 2 diabetes, hypertension, and cardiovascular disease (CVD) [7–9]. Longitudinal and meta-analytic evidence suggests that short sleep increases the incidence and severity of CVD, CVD comorbidities, and risk of CVD mortality [7, 10]. Too much sleep has been linked to poor health outcomes as well, with longer sleep duration associated with higher rates of health concerns such as inflammation, CVD, and depression [7, 11, 12]. Therefore, either extreme of sleep duration (short or long) is associated with higher risk for various health outcomes. Other health concerns, such as depression, may have independent effects on both sleep and health [11], thus introducing complex, reciprocal, and interacting relationships between sleep and various health outcomes.

While sleep patterns tend to shift for all people across the lifespan, older women are more likely to report poor sleep quality, restless sleep, and more nocturnal awakenings than any other subgroup of the population [13, 14]. The 2005 NSF Sleep in America poll also revealed that women in general are more likely than men to have difficulty falling and staying asleep and to experience more daytime sleepiness [15]. Interestingly, a large Finish cohort study found that sleep duration is an independent risk factor for cardiovascular mortality and morbidity in women but not in men [12]. Across the lifespan women report more sleep disturbances than men, with increases in reported difficulties during the menopausal transition [14, 16]. This is a time period where significant physiological changes and symptoms (e.g. hot flashes) occur that may exacerbate or instigate sleep disturbances [14, 16] and may present an opportunity for intervention with a high risk population for sleep disturbances.

Due to the wealth of evidence linking sleep to a range of health outcomes, there is growing recognition of the importance of sleep to improve population health [7–9]. Pharmacological approaches have been a primary antidote to these problems, yet carry with them common side effects and are not always effective [17]. Cognitive Behavioral Therapy, bright light therapy, and physical activity are non-pharmacological approaches that may help to improve sleep quality and duration for older adults [17]. Physical activity, for example walking, represents an intervention that can be done at little to no cost, without the need for trained professionals, and results in a range of health benefits in addition to potential improvements in sleep health. Indeed, physically active women seem to sleep more and better than sedentary women [18]. Further, research shows that women are more likely to engage in behavioral approaches to improve sleep [19], such as physical activity, which may provide feasible and effective approaches to address this public health concern.

Studies evaluating the effects of physical activity (PA) interventions on sleep quality and duration, though limited, suggest PA can improve overall sleep quality [20]. In a systematic review of 6 trials, the authors found that compared to the control group, participants in exercise interventions reported improved global sleep scores and reduced sleep latency and medication use; there were no differences between groups in other sleep outcomes, such as sleep duration, sleep efficiency, or sleep disturbance [20]. Other recent research continues to suggest some improvement in sleep outcomes with a range of exercise interventions [21–26] for different adult and patient populations. In a randomized controlled trial with peri- and post-menopausal women, a brief moderate-intensity exercise intervention (3 times per week for 12 weeks) was associated with some improvements in self-reported sleep quality and insomnia symptoms [26]. However, the clinical experimental studies to date may not translate into real world interventions in large populations.

There are only a few studies investigating the real world daily relations between PA and sleep through objective assessments [27–30]. In studies with adult populations, there was consistency in poor sleep at night predicting lower levels of PA the following day in three studies [27–29], but there was no significant effect in a fourth study [30]. PA predicting sleep that night was less consistent. One study found more PA was related to less total sleep the night following activity and unrelated to other measures of sleep health [29]. However, another study found greater PA was associated with better self-report sleep quality ratings and reduced wake time after sleep onset (WASO) the subsequent night [28]. Two studies found no relation between daily PA and subsequent sleep [27, 30]. There are numerous factors that may contribute to the inconsistencies in the few studies conducted to date including differences in the exercise protocols studied, such as the intensity and duration of programs, and their interactions with individual characteristics such as fitness, age, and gender [31]. Questions remain over the sleep and health benefits associated with specific levels of physical activity (light, moderate, vigorous) and at what combinations or thresholds, and differences in effects across individuals and across the lifespan. Such nuanced information is needed in order to tailor recommendations to promote optimal health.

Exercise is implicated in a range of physiological changes, including potential alterations of circadian rhythms [32]. Circadian rhythms are known to weaken and shift across the lifespan, thus providing a potential underlying contributor to changes in sleep for older adults [3, 33]. Several studies have described a time-dependent exercise-induced phase shift of the onset of melatonin [34]. Under laboratory conditions, morning exercise enhanced parasympathetic activity as indicated by heart rate variability, whereas evening exercise increased heart rate the following night and delayed the circadian rhythm by about 1 hour [35]. A study using national survey data found that morning exercisers reported the best sleep health overall; though those with PA fewer than 4 hours before sleep did not vary in sleep quality from non-exercisers [36]. Fairbrother and colleagues [37] found that a bout of moderate-intensity aerobic exercise in the morning (7 AM) resulted in the most beneficial responses in blood pressure and improved overall sleep quality compared to exercise in the afternoon (1 PM) or evening (7 PM). Overall, morning PA seems to be supported in the current literature as beneficial for overall sleep health.

Other factors to consider include time outdoors and light exposure as potentially influencing both PA levels and sleep quality [32]. Bright light therapy, much like spending time outdoors, is thought to influence circadian rhythms and associated physiological processes [38]. During winter months, bright light therapy is recommended to combat fatigue and emotional disturbances that accompany limited light exposure in some groups [38]. Additionally, exercise is positively linked to outdoor time, with individuals spending more time outdoors being more physically active [39, 40]. Therefore, these variables may be interrelated and benefit from simultaneous evaluation. It is only recently that objective measures, such as Global Positioning System (GPS) devices, have been validated to evaluate time spent outdoors [41].

This is one of the first studies to examine the interrelation between sleep, timing of daily PA, and time spent outdoors measured objectively over a consecutive 7-day period by accelerometers and GPS devices. Following the absence of a main effect for sleep and PA [30], this research explores a potential moderator of the relation between sleep and PA: time spent outdoors. There were 2 main hypotheses: (1) people with more time outdoors would have a stronger relation between PA and sleep (i.e. for people with higher levels of time outdoor, there would be a stronger positive relation between PA and sleep than for people with lower levels of time outdoors); and, (2) those with more time outdoors in the morning (as opposed to the afternoon) would have better sleep health. Sleep health in this study is measured by accelerometer-derived scores of total sleep time, sleep efficiency, sleep latency, and wake after sleep onset times.

## Materials and methods

A total of 377 women (mean age 55.31 ± 10.17) from studies conducted across four sites in the U.S. (UC San Diego, Washington University in St. Louis, Harvard University, and University of Pennsylvania) were included in the current analyses supported by the Transdisciplinary Research on Energetics and Cancer (TREC) initiative [42]. Participants were recruited from existing TREC studies, which included varying recruitment strategies (local community samples and national U.S. samples) and methods (observational and intervention studies). Some participants were specifically recruited as breast cancer survivors or nurses. In some instances, participants screened as ineligible for the TREC intervention studies were contacted for the current study.

All participants completed a written consent form prior to participating in the study, and all participants completed additional measures using methods that were standardized across sites. All sites were trained extensively on data collection, participant compliance, and data screening techniques. Therefore, this is a convenience sample that completed standardized measures with common inclusion criteria, which enabled pooling of the data. The UC San Diego Human Research Protections Program provided human research ethical clearance for the project, and research ethics approval was ascertained at each participating institution as well. Eligibility criteria were: female, 21 to 75 years old, self-reported BMI between 21.0 and 39.9, ability to ambulate unassisted, not pregnant or breast-feeding, and willing to wear monitoring devices for 7 days. Therefore, no men were recruited as part of the current study.

### Measures

Participants were enrolled in the study across the 4 sites over the course of the 12-month calendar year. Participants completed surveys and wore objective measurement devices over at least 5 consecutive days and nights in combination with self-reported sleep logs reporting the times they were in and out of bed for the corresponding sleep periods.

#### Sleep

Measures of sleep quality and duration were assessed using wrist-worn accelerometry (ActiGraph GT3X+; ActiGraph, LLC; Pensacola, FL). The GT3X+ has been recently validated for sleep-wake estimation against polysomnography [43]. Scores were calculated for Total Sleep Time (TST), Sleep Efficiency (SE), Sleep Onset Latency (SOL), and WASO. Minute-level data were scored with ActiLife using validated algorithms [44], which assigned a sleep or wake score for each minute. Total sleep time was the sum of the number of sleep minutes during the sleep period and sleep-efficiency was the percentage of time spent in bed that the person was asleep. Sleep latency scores reflected the number of minutes it took the person to fall asleep once in bed and WASO was the number of minutes until the first awakening after sleep onset. Individuals kept daily logs (completed upon arising) of the times they got into and out of bed, which were used to calculate the time in bed. The self-report log was used to define the in-bed and out-of-bed times used by the algorithm to calculate the sleep variables. Number of nights of sleep was dependent on the number of days of physical activity, as described below.

#### Physical activity

Physical activity was estimated through hip-worn accelerometers (Actigraph GT3X+). Participants were given the device to wear for 7 consecutive days. In order for participant data to be considered valid, the participant had to either (a) wear the device for at least 10 hours per day for at least 5 days during the 7-day period; or, (b) wear the device for at least 3000 total minutes across four days or fewer. Time periods with 90 consecutive minutes of zero counts of activity were considered non-wear time [45]. A sum score was generated to represent the total number of minutes spent in moderate-to-vigorous physical activity (MVPA). A cut point of 1041 counts per minute was employed to assess minutes in MVPA that were performed at a moderate intensity or greater, as appropriate for this age group [46].

#### Outdoor time

GPS data were used to calculate daily time spent outdoors, and outdoor PA minutes. Participants wore a Qstarz BT1000X GPS device. A GPS device logged X,Y location coordinates, distance, speed, elevation, and time. The Qstarz has an accuracy of 3 m and recorded location every 15 s. We configured the device to record additional satellite information (Signal to Noise Ratio: SNR), and applied validated cut points to detect outdoor locations; the less noise, the more likely the participant is outdoors [41]. Sum scores were generated to represent total time spent outdoors.

#### Time of day

Time of day was classified to reflect the time of measurement, either as morning (6 am to 11:59 am) or afternoon (12 pm to 5:59 pm). Sum scores were generated to represent total minutes spent outdoors in either the morning or afternoon.

#### Season

The seasons were defined according to meteorological seasons in the U.S. as: Winter (December, January, February; *n* = 90), Spring (March, April, May; *n* = 123), Summer (June, July, August; n = 36), and Fall (September, October, November; *n* = 103). Participants were allocated a season of wear based on the dates in their accelerometer files.

#### Demographics

Demographic data were collected from all participants through self-report measures, including age, race/ethnicity, education, marital status, and employment status (full time, part time, or not employed). Participants provided a general rating of self-reported health status on a 5-point Likert scale (poor to excellent), with higher scores indicating greater self-reported health. Participants’ self-reported height and weight was used to calculate Body Mass Index (BMI; kg/m^2^) using standard procedures. Demographic and health indicators were included as covariates in the models.

### Preliminary analyses

Accelerometer-measured sleep outcomes (e.g., Total Sleep Time, WASO), MVPA and outdoor time were calculated for each day across the week. The daily minutes of MVPA and outdoor time were matched to subsequent nightly sleep quality outcomes, creating temporally linked day-level dataset. If the day was missing MVPA or sleep information it was removed from the analysis.

#### Hypothesis 1: People with more time outdoors will have a stronger relation between sleep and MVPA

We used linear mixed models (LMMs), with a random intercept, for continuous outcomes to examine the interaction between daily outdoor time and PA with daily sleep outcomes (TST, SE, SOL, WASO), adjusting for age, BMI, self-reported health, employment status, education, marital status, race, and device wear time. Due to normality violations and skew for the SE variable, the log of the inverted scale was calculated for analyses including that variable. SOL and WASO were log transformed. In addition, personal averages for physical activity and outdoor time [person-centered] variables were included in the models to separate the between-subject variation and the within-subject variation, in order to interpret the moderation of the association between daily physical activity and sleep by daily outdoor time after adjusting for person-level effects. We tested potential moderating effects of BMI, age, employment category, and season of data collection, all of which were not significant. Therefore, the interaction terms were not included for those variables in the final models.

#### Hypothesis 2: People with more time outdoors in the morning (as opposed to the afternoon) will have improved sleep health

We used separate (LMMs), with a random intercept, to examine the association between outdoor time of day and sleep outcomes. Each model included the total minutes of outdoor time accumulated in the morning and afternoon and adjusted for age, BMI, self-reported health, employment status, education, marital status, race, and device wear time. SE, SOL and WASO variables were transformed as described above and participant averaged outdoor time of day variables were added to the models to adjust for between-subject effects. The general multilevel modeling equations for hypotheses 1 and 2 are provided in S1 Table.

## Results

Table 1 outlines the study sample characteristics including demographics, sleep, and PA descriptive statistics for the final sample included in the analyses. The average number of days and nights worn for all devices was 5.5. Devices were worn throughout the year. Matched GPS and accelerometer data meeting wear time criteria were available on 352 of the 377 women recruited. There was no significant difference in characteristics of the women with and without available data. The majority of the women in the study were White (77.2%), with an average BMI of 28.3 (SD = 6.3). They had 3 chronic conditions on average, including surviving breast cancer. The sample slept an average of 6.8 hours per night (SD = 0.96), and had an average sleep efficiency of 85.7% (SD = 7.2%), sleep latency of 7.8 minutes (SD = 8.2), and WASO of 61.4 minutes (SD = 34.2). Participants averaged 64.6 minutes (SD = 34.1) of MVPA per day using 1041 counts per minute as the cut-off for moderate to vigorous intensity activity. Participants spent on average 146 minutes outdoors per day. The absence of a main effect relationship between PA and sleep is reported elsewhere [30].

**Table 1:** Demographic, physical activity, and sleep characteristics of the adult female convenience sample (N = 352).

Variable		M/*n*	SD/*percentage*
Age		55.4	10.2
Marital status (married or living with partner)		249	70.2%
Education	Grade school HS diploma/GED	33	9.4%
Some college or Associate Degree		71	20.2%
College graduate		119	33.8%
Graduate degree (Master's, Ph.D., M.D., J.D., etc.)		123	34.9%
Employment status			
Employed full time 35 hours or more per week		174	49.4%
Employed part time less than 35 hours per week		80	22.7%
All others: (Employed in seasonal labor, Out of work/looking for work, Homemaker, Retired, Do not work or unable to work)		98	27.8%
Race/ethnicity (White)		272	77.2%
BMI		28.34	6.3
Self-reported health		3.6	0.9
Device wear time (minutes)		865.4	82.5
Total Sleep Time (TST) (hours)		6.8	0.96
Sleep Efficiency (SE)		85.7%	7.22%
Number of days with 85% or higher sleep efficiency		3.6	2.1
Sleep Latency		7.8	8.2
Wake After Sleep Onset (WASO) (minutes)		61.4	34.2
Total outdoor time (minutes)		146.4	107.80
Season of data collection (% of days in each season)	Winter	90	25.6%
	Fall	103	29.3%
	Spring	123	35.9%
	Summer	36	10.2%
Moderate-to-vigorous physical activity (MVPA) per day		64.6	34.08

Note. The final sample for subsequent analyses included 352 participants due to missing sleep and accelerometry wear-time requirements.

### Hypothesis 1: People with more time outdoors will have a stronger relation between sleep and MVPA

Table 2 provides the results for associations between key variables and sleep outcomes (S2 Table provides output for the full model). There was a significant interaction (S1 Fig provides a visual representation of the interaction) between time spent outdoors and MVPA in predicting Total Sleep Time (*p* = 0.04). For people with less time outdoors (-1 SD), for every 1-hour increase of MVPA, Total Sleep Time decreased by 8.5 minutes per day. However, for people with the greatest amount of time spent outdoors (+1 SD), for every 1-hour increase in MVPA, Total Sleep Time increased by 1.6 minutes per day. The interaction between MVPA and outdoor time was not significant for Sleep Efficiency (*p* = 0.14), WASO (*p* = 0.14), nor Sleep Latency (*p* = 0.76). There were no significant between person effects for the average hours per day of MVPA or time outdoors in predicting sleep outcomes.

**Table 2:** The moderation of the relationship between Moderate to Vigorous Physical Activity (MVPA) and sleep by outdoor time.

	Total Sleep Time (hours/day)^†^		Sleep Efficiency (%)^†^		Latency (minutes)^†^		Wake after Sleep Onset (minutes)^†^	
	β	95% CI	exp(β)*	95% CI	exp(β)*	95% CI	exp(β)*	95% CI
MVPA (1041+ counts per minute; hours/day)	-0.150	(-0.305, 0.005)	-0.043	(-0.107, 0.018)	0.118	(-0.054, 0.322)	0.039	(-0.046, 0.131)
Outdoor time (hours/day)	-0.043	(-0.098, 0.013)	-0.029	(-0.051, -0.007)	0.011	(-0.048, 0.073)	0.040	(0.009, 0.072)
MVPA (h/d) & Outdoor Time (hours/day) Interaction^‡^	0.039	(0.003, 0.075)	0.011	(-0.003, 0.024)	-0.006	(-0.043, 0.032)	-0.015	(-0.034, 0.005)
MVPA (pt. avg; hours/day) ^§^	-0.103	(-0.308, 0.102)	-0.027	(-0.107, 0.018)	-0.009	(-0.177, 0.194)	0.029	(-0.082, 0.154)
Outdoor Time (pt. avg; hours/day) ^§^	0.003	(-0.065, 0.071)	0.007	(-0.022, 0.034)	0.018	(-0.044, 0.084)	-0.013	(-0.05, 0.024)

* These values should be interpreted as the percent change in Y for every unit change in X.

^†^ Models adjusted for age, body mass index (BMI), employment status, education, marital status, race, self-reported health, and device wear time.

^‡^ The interaction term estimates the magnitude of the difference in the relation between sleep quality and physical activity for each increment of outdoor time.

^§^ pt. avg; hours/day = Participant average (hours per day) and should be interpreted as between person effects.

### Hypothesis 2: People with more time outdoors in the morning (as opposed to the afternoon) will have improved sleep health

Table 3 provides the results for associations between key variables and sleep outcomes (S3 Table provides output for the full model). People who on average had higher time outdoors in the afternoon had greater total sleep time (*p =* 0.02). However, when examining within person variability over the week and after adjusting for time outdoors in the morning, the association between Total Sleep Time and time outdoors in the afternoon was not significant (*p* = 0.16). The main within person effect for time outdoors in the morning approached significance in predicting Total Sleep Time (*p* = 0.06) after adjusting for time outdoors in the afternoon. For within person Sleep Efficiency, the association with time outdoors in the afternoon was significant after adjusting for morning hours outdoors (*p* = 0.01). People with more time outdoors in the afternoon had the lowest sleep efficiency ratings. Time outdoors in the morning was not significant in predicting sleep efficiency (*p* = 0.80) when adjusting for afternoon hours outdoors. Neither time outdoors in the morning or afternoon had a significant association with Sleep Latency or WASO.

**Table 3:** The association between time of day outdoors and sleep.

	Total Sleep Time (hours/day)^†^		Sleep Efficiency (%)^†^		Latency (minutes)^†^		Wake after Sleep Onset (minutes)^†^	
	β	95% CI	exp(β)*	95% CI	exp(β)*	95% CI	exp(β)*	95% CI
Morning Outdoor Time (daily hours)	0.094	(-0.003, 0.191)	-0.005	(-0.043, 0.032)	0.027	(-0.079, 0.144)	0.027	(-0.026, 0.083)
Afternoon Outdoor Time (daily hours)	-0.052	(-0.124, 0.021)	-0.036	(-0.066, -0.008)	-0.012	(-0.089, 0.072)	0.035	(-0.005, 0.078)
Morning Outdoor Time (pt. avg hours/day) ^§^	-0.207	(-0.427, 0.0121)	0.038	(-0.055, 0.124)	-0.112	(-0.268, 0.078)	-0.073	(-0.179, 0.047)
Afternoon Outdoor Time (pt. avg hours/day) ^§^	0.205	(0.033, 0.378)	-0.023	(-0.101, 0.049)	0.111	(-0.044, 0.292)	0.054	(-0.042, 0.16)

* These values should be interpreted as the percent change in Y for every unit change in X.

^†^ Models adjusted for age, body mass index (BMI), employment status, education, marital status, race, self-reported health, and device wear time.

^§^ pt. avg = Participant average (hours per day) and should be interpreted as between person effects.

## Discussion

To our knowledge this was the first study to employ objective 24-hour accelerometer and daytime GPS data to assess the effects of outdoor time, time of day, MVPA, and their interactions on sleep health. Evidence demonstrates that PA may improve sleep health and additional studies have suggested that light exposure may be related to sleep. However, specific recommendations around when or where to be active for sleep health are not yet available. We found significant moderation by outdoor time of the relationship between MVPA and total sleep time. We found that women with less total time outdoors had shorter nightly sleep as their MVPA increased as estimated objectively by GPS and hip and wrist worn accelerometers. We also found worse sleep efficiency among women when they had more afternoon time outdoors. However, women who on average had more time outdoors in the afternoon had more total sleep time than women who had less afternoon outdoor time on average. The effect for sleep efficiency, while not significant, was in the expected direction, with women who had more time outdoors in the afternoon having worse sleep efficiency. Therefore, it may be that women may spend more time in bed but have less efficient sleep when they spend more time outdoors in the afternoon. Alternatively there may be individual variability in the impact of time of day outdoors for different individuals.

Overall, these findings suggest some within and between person differences and demonstrate some consistency with the biological mechanisms proposed to explain the relation between light exposure, MVPA, and sleep health. Both physical activity and the aging process can affect circadian rhythms and thus be contributing factors in the rises in sleep disturbances experienced by older adults (see [33] for a review). Light exposure and physical activity are two factors that provide direct feedback to the circadian system, which may have resulted in the short-term effects identified in this research. These and other findings might suggest a potential mediating pathway whereby exercise performed outside at certain times of day may engage a specific pathway (i.e. UV exposure and circadian rhythms) that may affect sleep health independent of other pathways (e.g. thermoregulation, autonomic arousal). There may be an additive effect of light and exercise on phase shifting/circadian rhythm [47]. Research has demonstrated that natural sunlight (exposed when being outside) is a stronger cue for the internal clock than are electric lights [48]. Therefore, exercise and time outdoors may occur in ways that are mutually beneficial or in ways that may send different feedback to the circadian system, depending on where, when, and how much of each are experienced.

Those with sleep problems might consider getting their outdoor time and light exposure during the morning (but not early morning if struggling with advanced sleep phase) as opposed to afternoon hours, which is consistent with recommendations for bright light therapy [38]. Morning exercisers might also have more consistent daily routines, which have been linked to fewer self-reported sleep problems [49] and better functioning circadian systems [33]. Further studies should assess the clinical relevance of the time of day, amount of time spent outdoors, and amount of physical activity to develop more precise recommendations to optimize sleep health. While it cannot be tested in the current data, the long-term benefits of outdoor PA interventions may be even greater with the reduction of comorbidities that contribute to rising sleep disturbances in aging.

There are several limitations to this study. The analytic sample included 352 healthy, primarily White, fairly active, middle aged to older women from across the U.S. studied over 5 days. These analyses may not generalize to other populations including younger women or men. Associations may also be stronger in populations with more variation in sleep quality than detected in this sample. Further associations should be tested in prospective studies and in interventions. In this study, we only evaluated the acute effects over a 5-day period of time, therefore longer-term effects cannot be assessed. It may be that the acute effects of MVPA are less effective in promoting sleep quality, but that over time the benefits of regular MVPA on physiological functioning promote longer-term sleep health. Given the multiple physiological pathways and bidirectional effects that underlie the PA-sleep relationship [50] further research is needed. We did not look at the relation between PA and sleep with health outcomes, such as depression, inflammation, and CVD, which may introduce their own direct and indirect influences on sleep. Research is needed to test causal mechanisms and to tease apart the multiple influences on sleep health in the aging process.

We utilized a cut-off score of 1041 counts per minute to capture MVPA as opposed to all activity (including low intensity) within a day. Other studies have utilized different cut-off scores, such as Lambiase and colleagues [29] who used a cut-off of 760 counts per minute, albeit with an older sample (M age = 73 years) to categorize MVPA. It may be that the PA-sleep relationship also varies dependent on the intensity of activity. A recent study by Tsunoda and colleagues [51] found differences in the intensity of activity in predicting reports of sleep sufficiency (Assessed by the item: Do you sleep well and get a sufficient amount of rest?). Middle-aged adults (Mean age = 45.5 years) engaging in moderate high intensity exercise, and older adults (Mean age = 65.3 years) engaging in moderate low intensity exercise were less likely to report insufficient sleep [51]. Further research is needed to examine this relationship across the lifespan, for men and women, and the potential differences dependent on a person’s level of fitness.

While we used validated objective measures of sleep, MVPA, and outdoor location, the algorithms applied to classify these behaviors and locations may contain some measurement error. In particular, outdoor location is merely a proxy for light exposure. Other wearable devices (e.g. Actiwatch Spectrum PLUS) may prove useful measures of light exposure. In one study lux readings from an accelerometer were associated with GPS measured outdoor time [52]. However, the use of objective measures of the primary variables of interest is an important contribution of this research. Additionally, the sample was geographically diverse and represented a group at risk for developing CVD and other sleep-related health outcomes, and more likely to engage in behavioral interventions for sleep disturbances [19].

This study suggests that morning outdoor MVPA may confer sleep efficiency benefits. Given the multiple health benefits of MVPA and that outdoor MVPA may be more enjoyable and endure for longer periods, it seems reasonable to encourage outdoor MVPA to also support improved sleep health. Accumulating evidence for the benefits of outdoor PA [39, 40, 53] may help support efforts to develop policies around the provision of safe outdoor spaces for PA in all age groups [54].

## Supporting information

S1 Table General multilevel modeling equations utilized in testing the hypotheses. (PDF)

S2 Table The interaction of outdoor time on the relation between Moderate-to-Vigorous Physical Activity (MVPA) and sleep. (PDF)

S3 Table The relation between morning and afternoon outdoor time and sleep. (PDF)

S1 Fig Moderating effect of outdoor time on the relation between total sleep time and Moderate to Vigorous Physical Activity (MVPA) among older adult women. (PDF)

## References

[1] Ram S, Seirawan H, Kumar SK, Clark GT. Prevalence and impact of sleep disorders and sleep habits in the United States. Sleep Breath. 2010; 14: 63–70. doi: 10.1007/s11325-009-0281-3 19629554 10.1007/s11325-009-0281-3

[2] Crowley K. Sleep and sleep disorders in older adults. Neuropsychol Rev. 2011; 21: 41–53. doi: 10.1007/s11065-010-9154-6 21225347 10.1007/s11065-010-9154-6

[3] Neikrug AB, Ancoli-Israel S. Sleep disorders in the older adult–a mini-review. *Gerontology*. 2009; 56: 181–189. doi: 10.1159/000236900 19738366 10.1159/000236900 PMC2842167

[4] Moraes W, Piovezan R, Poyares D, Bittencourt LR, Santos-Silva R, Tufik S. (2014). Effects of aging on sleep structure throughout adulthood: a population-based study. *Sleep Med*. 2014; 15: 401–409. doi: 10.1016/j.sleep.2013.11.791 24657204 10.1016/j.sleep.2013.11.791

[5] Lauderdale DS, Schumm LP, Kurina LM, McClintock M, Thisted RA, Chen JH, Waite L. Assessment of sleep in the national social life, health, and aging project. J Gerontol B Psychol Sci Soc Sci. 2014; 69(Suppl 2): S125–S133. 25360013 10.1093/geronb/gbu092 PMC4303091

[6] Wolk R, Gami AS., Garcia-Touchard A, Somers VK. Sleep and cardiovascular disease. Curr Probl Cardiol. 2005;30: 625–662. doi: 10.1016/j.cpcardiol.2005.07.002 16301095 10.1016/j.cpcardiol.2005.07.002

[7] Cappuccio FP, Cooper D, D'Elia L, Strazzullo P, Miller MA. Sleep duration predicts cardiovascular outcomes: a systematic review and meta-analysis of prospective studies. Eur Heart J. 2011; 32: 1484–1492. doi: 10.1093/eurheartj/ehr007 21300732 10.1093/eurheartj/ehr007

[8] Cappuccio FP, D'Elia L, Strazzullo P, Miller MA. Quantity and quality of sleep and incidence of type 2 diabetes: a systematic review and meta-analysis. Diabetes Care. 2010; 33: 414–420. doi: 10.2337/dc09-1124 19910503 10.2337/dc09-1124 PMC2809295

[9] Cappuccio FP, Taggart FM, Kandala NB, Currie A, Peile E, Stranges S, et al Meta-analysis of short sleep duration and obesity in children and adults. Sleep, 2008;31: 619–626. 18517032 10.1093/sleep/31.5.619 PMC2398753

[10] Jackson CL, Redline S, Emmons KM. Sleep as a potential fundamental contributor to disparities in cardiovascular health. Annu Rev Public Health. 2015;36: 417–440. doi: 10.1146/annurev-publhealth-031914-122838 25785893 10.1146/annurev-publhealth-031914-122838 PMC4736723

[11] Mezick EJ, Hall M, Matthews KA. Are sleep and depression independent or overlapping risk factors for cardiometabolic disease? Sleep Med Rev. 2011; 15: 51–63. doi: 10.1016/j.smrv.2010.03.001 20494595 10.1016/j.smrv.2010.03.001 PMC2928398

[12] Kronholm E, Laatikainen T, Peltonen M, Sippola R, and Partonen T. Self-reported sleep duration, all-cause mortality, cardiovascular mortality and morbidity in Finland. Sleep Med. 2011; 12: 215–221. doi: 10.1016/j.sleep.2010.07.021 21317033 10.1016/j.sleep.2010.07.021

[13] Nowakowski S, Meers J, Heimbach E. Sleep and Women's Health. Sleep Med Res. 2013; 4: 1–22. 25688329 10.17241/smr.2013.4.1.1 PMC4327930

[14] Xu Q, Lang CP, Rooney N. A systematic review of the longitudinal relationships between subjective sleep disturbance and menopausal stage. Maturitas. 2014; 79: 401–412. doi: 10.1016/j.maturitas.2014.09.011 25449825 10.1016/j.maturitas.2014.09.011

[15] National Sleep Foundation [Internet]. Women and sleep. https://sleepfoundation.org/sleep-topics/women-and-sleep

[16] Bruyneel M. Sleep disturbances in menopausal women: Aetiology and practical aspects. Maturitas, 2015; 81: 406–409. doi: 10.1016/j.maturitas.2015.04.017 26002789 10.1016/j.maturitas.2015.04.017

[17] Montgomery P, Dennis J. A systematic review of non-pharmacological therapies for sleep problems in later life. Sleep Med Rev. 2004; 8: 47–62. doi: 10.1016/S1087-0792(03)00026-1 15062210 10.1016/S1087-0792(03)00026-1

[18] De Castro Toledo Guimaraes LH, de Carvalho LB, Yanaguibashi G, do Prado GF. Physically active elderly women sleep more and better than sedentary women. Sleep Med. 2008; 9: 488–493. doi: 10.1016/j.sleep.2007.06.009 17765012 10.1016/j.sleep.2007.06.009

[19] Venn S, Meadows R, Arber S. Gender differences in approaches to self-management of poor sleep in later life. Soc Sci Med. 2013;79: 117–123. doi: 10.1016/j.socscimed.2012.09.037 23141555 10.1016/j.socscimed.2012.09.037

[20] Yang PY, Ho KH, Chen HC, Chien MY. Exercise training improves sleep quality in middle-aged and older adults with sleep problems: a systematic review. J Physiother. 2012;58: 157–163. doi: 10.1016/S1836-9553(12)70106-6 22884182 10.1016/S1836-9553(12)70106-6

[21] Du S, Dong J, Zhang H, Jin S, Xu G, Liu Z, et al Tai chi exercise for self-rated sleep quality in older people: a systematic review and meta-analysis. Int J Nurs Stud. 2015;52: 368–379. doi: 10.1016/j.ijnurstu.2014.05.009 24934815 10.1016/j.ijnurstu.2014.05.009

[22] Mustian KM, Sprod LK, Janelsins M, Peppone LJ, Palesh OG, Chandwani K, et al Multicenter, randomized controlled trial of yoga for sleep quality among cancer survivors. J Clin Oncol; 2013;31: 3233–3241. doi: 10.1200/JCO.2012.43.7707 23940231 10.1200/JCO.2012.43.7707 PMC3757292

[23] Passos GS, Poyares D, Santana MG, D’Aurea CVR, Youngstedt SD, Tufik S, et al Effects of moderate aerobic exercise training on chronic primary insomnia. Sleep Med. 2011;12: 1018–1027. doi: 10.1016/j.sleep.2011.02.007 22019457 10.1016/j.sleep.2011.02.007

[24] Sarris J, Byrne GJ. A systematic review of insomnia and complementary medicine. Sleep Med Rev. 2011;15: 99–106. doi: 10.1016/j.smrv.2010.04.001 20965131 10.1016/j.smrv.2010.04.001

[25] Viana VA, Esteves AM, Boscolo RA, Grassmann V, Santana MG, Tufik S, et al The effects of a session of resistance training on sleep patterns in the elderly. Eur J Appl Physiol. 2012;112: 2403–2408. doi: 10.1007/s00421-011-2219-2 22045416 10.1007/s00421-011-2219-2

[26] Sternfeld B, Guthrie KA, Ensrud KE, LaCroix AZ, Larson JC, Dunn AL, Anderson GL, Seguin RA, Carpenter JS, Newton KM, Reed SD. Efficacy of exercise for menopausal symptoms: a randomized controlled trial. *Menopause (New York*, *NY)*. 2014: 21; 330–338. 10.1097/GME.0b013e31829e4089 PMC3858421 23899828

[27] Baron KG, Reid KJ, Zee PC. Exercise to improve sleep in insomnia: exploration of the bidirectional effects. J Clin Sleep Med. 2013;9: 819–824. doi: 10.5664/jcsm.2930 23946713 10.5664/jcsm.2930 PMC3716674

[28] Dzierzewski JM, Buman MP, Giacobbi PR Jr, Roberts BL, Aiken-Morgan AT, Marsiske M, et al Exercise and sleep in community-dwelling older adults: evidence for a reciprocal relationship. J Sleep Res. 2014;23: 61–68. doi: 10.1111/jsr.12078 23980920 10.1111/jsr.12078 PMC3844037

[29] Lambiase MJ, Gabriel KP, Kuller LH, Matthews KA. Temporal relationships between physical activity and sleep in older women. Med Sci Sports Exerc. 2013;45: 2362–2368. doi: 10.1249/MSS.0b013e31829e4cea 23739529 10.1249/MSS.0b013e31829e4cea PMC3833970

[30] Mitchell JA, Godbole S, Moran K, Murray K, James P, Laden F, et al No evidence of reciprocal associations between daily sleep and physical activity. Med Sci Sports Exerc.2016; 48: 1950–1956. doi: 10.1249/MSS.0000000000001000 27285490 10.1249/MSS.0000000000001000 PMC5026562

[31] Driver HS, Taylor SR. Exercise and sleep. Sleep Med Rev. 2000;4: 387–402. doi: 10.1053/smrv.2000.0110 12531177 10.1053/smrv.2000.0110

[32] Buman MP, King AC. Exercise as a Treatment to Enhance Sleep. Am J Lifestyle Med. 2010;4: 500–514.

[33] Weinert D, Waterhouse J. The circadian rhythm of core temperature: effects of physical activity and aging. *Physiol Behav*. 2007; 90: 246–256. doi: 10.1016/j.physbeh.2006.09.003 17069866 10.1016/j.physbeh.2006.09.003

[34] Buxton OM, L'Hermite-Baleriaux M, Hirschfeld U, Cauter E. Acute and delayed effects of exercise on human melatonin secretion. J Biol Rhythms. 1997;12: 568–574. doi: 10.1177/074873049701200611 9406031 10.1177/074873049701200611

[35] Yamanaka Y, Hashimoto S, Takasu NN, Tanahashi Y, Nishide SY, Honma S, et al Morning and evening physical exercise differentially regulate the autonomic nervous system during nocturnal sleep in humans. Am J Physiol Regul Integr Comp Physiol.2015;309: R1112–21. doi: 10.1152/ajpregu.00127.2015 26333783 10.1152/ajpregu.00127.2015

[36] Buman MP, Phillips BA, Youngstedt SD, Kline CE, Hirshkowitz M. Does nighttime exercise really disturb sleep? Results from the 2013 National Sleep Foundation Sleep in America Poll. Sleep Med. 2014;15: 755–761. doi: 10.1016/j.sleep.2014.01.008 24933083 10.1016/j.sleep.2014.01.008

[37] Fairbrother K, Cartner B, Alley JR, Curry CD, Dickinson DL, Morris DM, et al Effects of exercise timing on sleep architecture and nocturnal blood pressure in prehypertensives. Vasc Health Risk Manag. 2014;10: 691–698. doi: 10.2147/VHRM.S73688 25540588 10.2147/VHRM.S73688 PMC4270305

[38] Pail G, Huf W, Pjrek E, Winkler D, Willeit M, Praschak-Rieder N, et al Bright-light therapy in the treatment of mood disorders. Neuropsychobiology. 2011;64: 152–162. doi: 10.1159/000328950 21811085 10.1159/000328950

[39] Kerr J, Marshall S, Godbole S, Neukam S, Crist K, Wasilenko K, et al The relationship between outdoor activity and health in older adults using GPS. Int J Environ Res Public Health. 2012;9: 4615–4625. doi: 10.3390/ijerph9124615 23330225 10.3390/ijerph9124615 PMC3546779

[40] Kerr J, Sallis JF, Saelens BE, Cain KL, Conway TL, Frank LD, et al Outdoor physical activity and self rated health in older adults living in two regions of the U.S. Int J Behav Nutr Phys Act. 2012;9: 4615–4625. 10.1186/1479-5868-9-89 PMC3464785 22846594

[41] Tandon PS, Saelens BE, Zhou C, Kerr J, Christakis DA. Indoor versus outdoor time in preschoolers at child care. Am J Prev Med. 2013;44: 85–88. doi: 10.1016/j.amepre.2012.09.052 23253655 10.1016/j.amepre.2012.09.052

[42] Patterson RE, Colditz GA, Hu FB, Schmitz KH, Ahima RS, Brownson RC, et al The 2011–2016 Transdisciplinary Research on Energetics and Cancer (TREC) initiative: rationale and design. Cancer Causes Control. 2013;24: 695–704. doi: 10.1007/s10552-013-0150-z 23378138 10.1007/s10552-013-0150-z PMC3602225

[43] Slater JA, Botsis T, Walsh J, King S, Straker LM, Eastwood PR. Assessing sleep using hip and wrist actigraphy. Sleep and Biological Rhythms. 2015;13: 172–180.

[44] Cole RJ, Kripke DF, Gruen W, Mullaney DJ, Gillin JC. Automatic sleep/wake identification from wrist activity. Sleep. 1992;15: 461–469. 1455130 10.1093/sleep/15.5.461

[45] Choi L, Liu Z, Matthews CE, Buchowski MS. Validation of accelerometer wear and nonwear time classification algorithm. Med Sci Sports Exerc. 2011;43: 357–364. doi: 10.1249/MSS.0b013e3181ed61a3 20581716 10.1249/MSS.0b013e3181ed61a3 PMC3184184

[46] Copeland JL, Esliger DW. Accelerometer assessment of physical activity in active, healthy older adults. J Aging Phys Act. 2009;17: 17–30. 19299836 10.1123/japa.17.1.17

[47] Youngstedt SD, Kline CE, Elliott JA, Zielinski MR, Devlin TM, Moore TA. (2016). Circadian phase-shifting effects of bright light, exercise, and bright light + exercise. J Circadian Rhythms. 2016;14: 1–8. 27103935 10.5334/jcr.137 PMC4834751

[48] Wright KP Jr, McHill AW, Birks BR, Griffin BR, Rusterholz T, Chinoy ED. Entrainment of the human circadian clock to the natural light-dark cycle. Curr Biol: CB. 2013; 23: 1554–1558. doi: 10.1016/j.cub.2013.06.039 23910656 10.1016/j.cub.2013.06.039 PMC4020279

[49] Monk TH, Reynolds CF III, Buysse DJ, DeGrazia JM, Kupfer DJ. The relationship between lifestyle regularity and subjective sleep quality. Chronobiol Int. 2003; 20: 97–107. 12638693 10.1081/cbi-120017812

[50] Chennaoui M, Arnal PJ, Sauvet F, Leger D. Sleep and exercise: A reciprocal issue? Sleep Med Rev. 2015;20: 59–72. doi: 10.1016/j.smrv.2014.06.008 25127157 10.1016/j.smrv.2014.06.008

[51] Tsunoda K, Kitano N, Kai Y, Uchida K, Kuchiki T, Okura T, Nagamatsu T. Prospective study of physical activity and sleep in middle-aged and older adults. Am J Prev Med. 2015; 48:662–673. doi: 10.1016/j.amepre.2014.12.006 25891000 10.1016/j.amepre.2014.12.006

[52] Tandon PS, Zhou C, Christakis DA. The frequency of outdoor play for preschool age children cared for at home-based child care settings. Acad Pediatr. 2012;12: 475–480. doi: 10.1016/j.acap.2012.06.010 22980727 10.1016/j.acap.2012.06.010

[53] Klinker CD, Schipperijn J, Kerr J, Ersboll AK, Troelsen J. Context-specific outdoor time and physical activity among school-children across gender and age: using accelerometers and GPS to advance methods. Frontiers in Public Health. 2014;2: 1–15. 24653983 10.3389/fpubh.2014.00020 PMC3949325

[54] Hunter RF, Christian H, Veitch J, Astell-Burt T, Hipp JA, Schipperijn J. The impact of interventions to promote physical activity in urban green space: a systematic review and recommendations for future research. Soc Sci Med. 2015;124: 246–256. doi: 10.1016/j.socscimed.2014.11.051 25462429 10.1016/j.socscimed.2014.11.051

