# Supplementary material for: The relations between sleep, time of physical activity, and time outdoors among adult women
Source: PLoS One. 2017 Sep 6;12(9):e0182013. doi: 10.1371/journal.pone.0182013 (PMC5587264; doi:10.1371/journal.pone.0182013)
Supplement: S1 Table — (PDF) [file pone.0182013.s001.pdf]

S1 Table. General Multilevel Modeling Equations Utilized in Testing the Hypotheses.

**Hypothesis 1**	For the ith day on the jth individual<br>Level-1 equation:<br>$Y_{ij} = \beta_{0j} + \beta_{1j} *MVPA_{ij} + \beta_{2j}*Outdoor_{ij} + \beta_{3j}*MVPA_{ij}*Outdoor_{ij} + \beta_{4j}*WearTime_{ij} + e_{ii}$<br>Level-2 equations (random intercept only):<br>$\beta_{0j} = \gamma_{00} + \gamma_{01}*MVPA_{j} + \gamma_{02}*Outdoor_{j} + \gamma_{03}*X_{1j} + \ldots + \gamma_{0p}*X_{0p} + u_{0j}$<br>where X_{1j} to X_{pj} represent the person-level covariates of age, BMI, employment status, education, marital status, race and self-reported health. $MVPA_j$ and $Outdoor_j$ represent mean values of MVPA and Outdoor time.<br>$\beta_{1j} = \gamma_{10}$; $\beta_{2j} = \gamma_{20}$; $\beta_{3j} = \gamma_{30}$; $\beta_{4j} = \gamma_{40}$<br>Reduced Form:<br>$Y_{ij} = \gamma_{00} + \gamma_{01}*MVPA_{j} + \gamma_{02}*Outdoor_{j} + \gamma_{03}*X_{1j} + \ldots + \gamma_{0p}*X_{0p} + \gamma_{10}*MVPA_{ij} + \gamma_{20}*Outdoor_{ij} + \gamma_{30}*MVPA_{ij}*Outdoor_{ij} + \gamma_{40}*WearTime_{ij} + e_{ii} + u_{0j}$
**Hypothesis 2**	For the ith day on the jth individual:<br>Level-1 equation:<br>$Y_{ij} = \beta_{0j} + \beta_{1j}$ Morning Outdoor $Time_{ij} + \beta_{2j}*$Afternoon Outdoor $Time_{ij} + \beta_{3j}*WearTime_{ij} + e_{ii}$<br>Level-2 equations (random intercept only):<br>$\beta_{0j} = \gamma_{00} + \gamma_{01}*$Morning Outdoor $Time_{j} + \gamma_{02}*$Afternoon Outdoor $Time_{j} + \gamma_{03}*X_{1j} + \ldots + \gamma_{0p}*X_{0p} + u_{0j}$<br>where X_{1j} to X_{pj} represent the person-level covariates of age, BMI, employment status, education, marital status, race and self-reported health. Morning Outdoor $Time_j$ and Afternoon Outdoor $Time_j$ represent mean values of morning and afternoon outdoor time<br>$\beta_{1j} = \gamma_{10}$; $\beta_{2j} = \gamma_{20}$; $\beta_{3j} = \gamma_{30}$<br>Reduced Form:<br>$Y_{ij} = \gamma_{00} + \gamma_{01}*$ Morning Outdoor $Time_{j} + \gamma_{02}*$ Afternoon Outdoor $Time_{j} + \gamma_{03}*X_{1j} + \ldots + \gamma_{0p}*X_{0p} + \gamma_{10}*$ Morning Outdoor $Time_{ij} + \gamma_{20}*$ Afternoon Outdoor $Time_{ij} + \gamma_{30}*WearTime_{ij} + e_{ii} + u_{0j}$
**Note.** Notations follow those proposed in Raudenbush, SW.; Bryk, AS. Hierarchical Linear Models. 2nd ed.Thousand Oaks, CA: Sage; 2002.	
