# Supplementary material for: The relations between sleep, time of physical activity, and time outdoors among adult women
Source: PLoS One. 2017 Sep 6;12(9):e0182013. doi: 10.1371/journal.pone.0182013 (PMC5587264; doi:10.1371/journal.pone.0182013)
Supplement: S2 Table — (PDF) [file pone.0182013.s002.pdf]

S2 Table. The Interaction of Outdoor Time on the Relation Between Moderate to Vigorous Physical Activity (MVPA) and Sleep.

	Total Sleep Time (hours/day)		Sleep Efficiency (%)		Latency (minutes)		Wake after Sleep Onset (minutes)	
	β	95% CI	exp(β)*	95% CI	exp(β)*	95% CI	exp(β)*	95% CI
MVPA (1041+ counts per minute; hours/day)	-0.150	(-0.305, 0.005)	-0.043	(-0.107, 0.018)	0.118	(-0.054, 0.322)	0.039	(-0.046, 0.131)
Outdoor time (hours/day)	-0.043	(-0.098, 0.013)	-0.029	(-0.051, -0.007)	0.011	(-0.048, 0.073)	0.040	(0.009, 0.072)
Daily hours of MVPA & Outdoor Time Interaction ‡	0.039	(0.003, 0.075)	0.011	(-0.003, 0.024)	-0.006	(-0.043, 0.032)	-0.015	(-0.034, 0.005)
MVPA (pt. avg; hours/day) †	-0.103	(-0.308, 0.102)	-0.027	(-0.12, 0.057)	-0.009	(-0.177, 0.194)	0.029	(-0.082, 0.154)
Outdoor time (pt. avg; hours/day) †	0.003	(-0.065, 0.071)	0.007	(-0.022, 0.034)	0.018	(-0.044, 0.084)	-0.013	(-0.05, 0.024)
Age [centered]	-0.010	(-0.021, 0.001)	-0.006	(-0.01, -0.001)	0.004	(-0.005, 0.013)	0.005	(-0.001, 0.011)
BMI [centered]	-0.014	(-0.031, 0.004)	-0.014	(-0.021, -0.006)	0.004	(-0.01, 0.018)	0.018	(0.008, 0.028)
Self-Reported Health [centered]	0.027	(-0.092, 0.145)	0.041	(-0.009, 0.089)	-0.088	(-0.173, 0.004)	-0.035	(-0.097, 0.031)
Employment (Ref: Employed 35+hrs/wk)								

Part time employed (< 35 hrs/wk)	0.072	(-0.169, 0.313)	-0.008	(-0.118, 0.092)	-0.097	(-0.258, 0.099)	0.059	(-0.074, 0.211)
Seasonal labor, out of work/looking, homemaker, retired & do not/unable to work	0.541	(0.3, 0.783)	0.093	(-0.006, 0.183)	-0.013	(-0.189, 0.201)	-0.020	(-0.143, 0.122)
Education (Ref: Grade school or some high school & High school diploma or G.E.D)								
Some college or Associate Degree	0.220	(-0.156, 0.596)	0.293	(0.169, 0.399)	-0.264	(-0.459, 0.002)	-0.344	(-0.468, -0.19)
College graduate	0.375	(0.024, 0.726)	0.329	(0.22, 0.424)	-0.259	(-0.444, -0.013)	-0.375	(-0.486, -0.24)
Graduate degree (Master's, Ph.D., M.D., J.D., etc.)	0.379	(0.023, 0.735)	0.342	(0.233, 0.436)	-0.292	(-0.47, -0.052)	-0.378	(-0.49, -0.242)
Marital status (Ref: married or living with partner)	-0.004	(-0.222, 0.213)	-0.007	(-0.106, 0.083)	-0.039	(-0.194, 0.147)	0.005	(-0.11, 0.134)
Race/ethnicity (Ref: White)	0.501	(0.26, 0.741)	0.236	(0.153, 0.312)	-0.163	(-0.312, 0.018)	-0.224	(-0.321, -0.112)
Hip Device Wear Time (minutes)	-0.131	(-0.168, -0.095)	0.012	(-0.002, 0.026)	-0.020	(-0.059, 0.021)	-0.032	(-0.051, -0.012)

* These values should be interpreted as the percent change in Y for every unit change in X

† pt. avg; hours/day = Participant average (hours per day) and should be interpreted as between person effects.

‡ The interaction term estimates the magnitude of the difference in the relation between sleep quality and physical activity for each increment of outdoor time.
