# Supplementary material for: The relations between sleep, time of physical activity, and time outdoors among adult women
Source: PLoS One. 2017 Sep 6;12(9):e0182013. doi: 10.1371/journal.pone.0182013 (PMC5587264; doi:10.1371/journal.pone.0182013)
Supplement: S3 Table — (PDF) [file pone.0182013.s003.pdf]

S3 Table. The Relation Between Morning and Afternoon Outdoor Time and Sleep.

	Total Sleep Time (hours/day)		Sleep Efficiency (%)		Latency (minutes)		Wake after Sleep Onset (minutes)	
	β	95% CI	exp(β)*	95% CI	exp(β)*	95% CI	exp(β)*	95% CI
Morning Outdoor Time (daily hours)	0.094	(-0.003, 0.191)	-0.005	(-0.043, 0.032)	0.027	(-0.079, 0.144)	0.027	(-0.026, 0.083)
Afternoon Outdoor Time (daily hours)	-0.052	(-0.124, 0.021)	-0.036	(-0.066, -0.008)	-0.012	(-0.089, 0.072)	0.035	(-0.005, 0.078)
Morning Outdoor Time (pt. avg hours/day) †	-0.207	(-0.427, 0.012)	0.038	(-0.055, 0.124)	-0.112	(-0.268, 0.078)	-0.073	(-0.179, 0.047)
Afternoon Outdoor Time (pt. avg hours/day) †	0.205	(0.033, 0.378)	-0.023	(-0.101, 0.049)	0.111	(-0.044, 0.292)	0.054	(-0.042, 0.16)
Age [centered]	-0.006	(-0.016, 0.004)	-0.005	(-0.01, -0.001)	0.003	(-0.006, 0.011)	0.005	(-0.001, 0.011)
BMI [centered]	-0.012	(-0.03, 0.005)	-0.013	(-0.021, -0.006)	0.002	(-0.012, 0.017)	0.018	(0.008, 0.028)
Self-Reported Health [centered]	0.016	(-0.102, 0.133)	0.039	(-0.011, 0.086)	-0.085	(-0.169, 0.007)	-0.034	(-0.095, 0.032)
Employment (Ref: Employed 35+hr/wk)								
Part time employed (Ref: < 35 hrs/wk)	0.056	(-0.187, 0.298)	-0.010	(-0.121, 0.09)	-0.075	(-0.24, 0.126)	0.054	(-0.079, 0.205)
Seasonal labor, out of work/looking, homemaker, retired & do not/unable to work	0.503	(0.258, 0.748)	0.102	(0.002, 0.192)	-0.016	(-0.193, 0.2)	-0.039	(-0.161, 0.101)

Education (Ref: Grade school or some high school & High school diploma or G.E.D)								
Some college or Associate Degree	0.265	(-0.114, 0.644)	0.305	(0.182, 0.41)	-0.296	(-0.483, -0.041)	-0.351	(-0.474, -0.199)
College graduate	0.376	(0.019, 0.732)	0.337	(0.227, 0.431)	-0.283	(-0.464, -0.041)	-0.382	(-0.492, -0.247)
Graduate degree (Master's, Ph.D., M.D., J.D., etc.)	0.386	(0.024, 0.748)	0.351	(0.241, 0.444)	-0.316	(-0.49, -0.081)	-0.386	(-0.498, -0.25)
Marital status (Ref: married or living with partner)	0.001	(-0.219, 0.221)	-0.013	(-0.113, 0.079)	-0.022	(-0.182, 0.168)	0.010	(-0.106, 0.141)
Race/ethnicity (Ref: White)	0.477	(0.236, 0.717)	0.236	(0.153, 0.311)	-0.165	(-0.313, 0.016)	-0.225	(-0.322, -0.114)
Hip Device Wear Time (minutes)	-0.140	(-0.177, -0.103)	0.009	(-0.005, 0.023)	-0.017	(-0.057, 0.024)	-0.031	(-0.05, -0.011)

*These values should be interpreted as the percent change in Y for every unit change in X.

† pt. avg = Participant average (hours per day) and should be interpreted as between person effects.
