# Supplementary material for: The relations between sleep, time of physical activity, and time outdoors among adult women
Source: PLoS One. 2017 Sep 6;12(9):e0182013. doi: 10.1371/journal.pone.0182013 (PMC5587264; doi:10.1371/journal.pone.0182013)
Supplement: S1 Fig — (PDF) [file pone.0182013.s004.pdf]

S1 Fig. Moderating effect of outdoor time on the relation between total sleep time and moderate to vigorous physical activity (MVPA) among older adult women

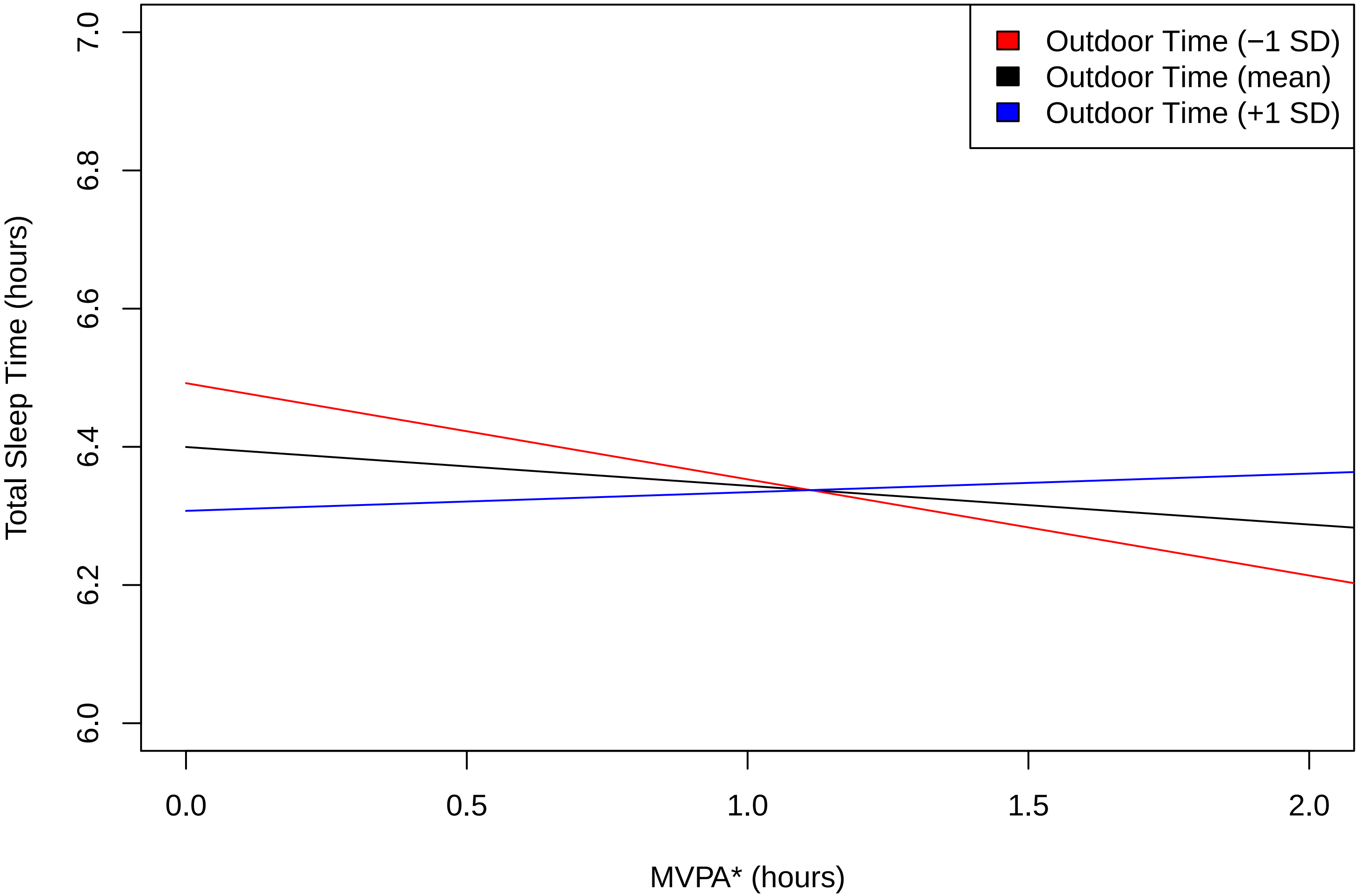

*MVPA: ≥ 1041 CPM
